# Prevalence of Acne Vulgaris in Adolescents and Young Adults in Al-Baha Region, Saudi Arabia

**DOI:** 10.7759/cureus.71293

**Published:** 2024-10-12

**Authors:** Aziz O Alsohaimi, Ahmed Alghamdi, Rajeh S Alghamdi, Abdullah H Alghamdi, Abdulaziz M Alkhathami, Mohammed A Alghamdi, Saeed A Alghamdi, Naif M ALZAHRANI, Nawaf S Alghamdi

**Affiliations:** 1 Department of Dermatology and Venereology, Faculty of Medicine, Al-Baha University, Al-Baha, SAU; 2 Department of Internal Medicine, Faculty of Medicine, Al-Baha University, Al-Baha, SAU

**Keywords:** acne vulgaris, acne-vulgaris, al-baha, al-baha region, cross section study, cross-section study, prevalence study, saudi arabia

## Abstract

Introduction

Acne vulgaris affects 9.4% of the global population, primarily targeting teenagers, and persists into adulthood. It is considered a major health challenge in Saudi Arabia due to its high prevalence, but there is a lack of studies in Al-Baha City, Saudi Arabia. Therefore, our goal is to estimate the prevalence of acne vulgaris in Al-Baha, which, in turn, may guide the education of those who are affected and help develop preventive measures.

Methodology

This is an observational cross-sectional study targeting adolescents and young adults in Al-Baha. The data was collected using an online version of a questionnaire designed to achieve the study’s objectives. Data was collected in Excel (Microsoft Corporation, Redmond, Washington, United States) and imported into R software version 4.2.2 (R Foundation for Statistical Computing, Vienna, Austria, https://www.R-project.org/).

Result

Approximately 70.2% (n=641) of the participants either currently have or have experienced acne, with the majority experiencing it on their faces (92%, n=451). The likelihood of experiencing acne was higher among individuals aged 23-25 years and females. The majority of participants assessed their acne as moderate (65%, n=292). Severe acne was associated with a higher risk of low mood and low self-esteem (p < 0.001).

Conclusion

Almost two-thirds of the participants (70.2%, n=641) reported either currently having or having experienced acne. Therefore, awareness should be raised about acne vulgaris and its management.

## Introduction

Acne vulgaris, a prevalent skin condition affecting 9.4% of the global population, primarily targets teenagers and persists into adulthood, affecting over 90% of men and 80% of women across all ethnicities. With an average onset age of 24 years, it stands as one of the most widespread skin disorders, with women being the most affected demographic [1,2].

Acne lesions include open and closed comedones, erythematous papules, pustules progressing to nodules, deep pustules, and pseudocysts in severe cases. Their development involves follicular hyperkeratinization, increased sebum production stimulated by androgen, colonization of hair follicles by Propionibacterium acnes, and subsequent immune and inflammatory reactions. Predominantly affecting the face, anterior chest, and upper back, acne’s pathogenesis is multifactorial [3,4].

Moderate to severe acne lesions contribute to decreased quality of life, lowered self-esteem, and decreased social interaction. Furthermore, they may induce anxiety, depression, and emotional distress, posing significant threats to overall well-being [5,6].

Acne vulgaris affects almost 70-80% of adolescents. A study reported that 71.23% of Peruvian students aged 17 years old have acne vulgaris. A higher prevalence rate was reported in multiple countries: 93.3% of Australian adolescents 16-18 years old and approximately 90% in Belgium and China. On the other hand, the prevalence rate in England is estimated to be approximately 50% [7-9].

According to a study in China, no cases of acne were observed in people who were younger than 10 years old, with only 1.6% of people aged 10 years having the disease. The incidence grew with age, reaching a peak of 46.8% by the time a person was 19 years old, and eventually became rare in those over 50. The study revealed that 68.4% of participants had mild acne, 26.0% had moderate acne, and 5.6% had severe acne. One out of three had received treatment previously, with a higher percentage among females (39.0%) compared to males (29.6%). Acne was found to be more common in adolescent males and adult females. Similar results were reported in two studies conducted in the United Kingdom [10,11].

In a study published in the Qassim region of Saudi Arabia, a significant percentage of teenagers held misconceptions about acne. The most prevalent misconception, held by 59.9% of the participants, was the belief that stress worsens acne [12]. In a separate study in Riyadh, Saudi Arabia, female patients were found to constitute around 68.2% of total cases of acne vulgaris. Additionally, it was reported that the average age of patients with acne vulgaris was 21 years, with the onset of the disease occurring at approximately 15 years old. On the other hand, a positive family history was reported in 42.5% of participants with the disease [13]. In a study conducted in Jeddah, Saudi Arabia (2017), it was found that the disease has a prevalence rate of 98% among females in medical school [14].

No previous study has been conducted in the Al-Baha region, Saudi Arabia, to determine the prevalence and psychological effects of acne vulgaris. Therefore, the aim of this study was to assess the acne vulgaris prevalence in adolescents and young adults in Al-Baha City, Saudi Arabia. The result of this study can be used to guide education for those who are affected and help develop preventive measures.

## Materials and methods

Study design and setting

This is an observational cross-sectional study that aims to assess the acne vulgaris epidemiology in young adults and adolescents in the Al-Baha region.

Study sample

The study targeted the adolescents and young adults of the Al-Baha region aged between 15 and 25. Any person outside of the Al-Baha region and/or outside of the age range was excluded from the study. To calculate the sample size, we initially determined the population size by searching the statistical database of the General Authority for Statistics in Saudi Arabia, which was 54918. Then, we built our sample size, which was 382 participants as a minimum, by using an online sample size calculator (Raosoft; Raosoft, Inc., Seattle, Washington, United States).

Study tool and data collection

The data were collected from July 15 to August 25 through an online questionnaire (see Appendices) that was designed to achieve the study’s objectives. The questionnaire was built based on multiple previous studies. [15-18] Questionnaire components include sociodemographic data, acne and its characteristics, associated factors, treatment behaviors, and the effect of the disease on quality of life and the psychology of patients.

Statistical analysis

Data was collected in Excel (Microsoft Corporation, Redmond, Washington, United States) and imported into R software version 4.2.2 (R Foundation for Statistical Computing, Vienna, Austria, https://www.R-project.org/). Descriptive statistics were used with numbers and percentages to represent categorical data and mean and standard deviation for continuous data. Chi-square and Fisher's exact tests were used to evaluate the determinants of experiencing acne, the association between acne severity and quality of life, and the association between treatment behavior towards acne and demographic characteristics. A p-value less than 0.05 was set as the significant level of the study.

Ethical considerations

The Scientific Research and Ethics Committee (REC) at the Faculty of Medicine at Al-Baha University approved the research with the approval number REC/MED/BU-FM/2023/69. We informed the participants that participation in the study was voluntary and that any information shared with us would be kept confidential and used only for research purposes. Access to the data was restricted to authorized researchers only.

## Results

The general characteristics of the six hundred forty-one participants showed that 56% (n=362) were aged 19-22 years, 54% (n=347) were females, 91% (n=582) were unmarried, and 55% (n=353) had bachelor's degrees, as shown in Table 1.

**Table 1:** General characteristics of the participants (N=293)

Characteristic	n (%)
Age	
15-18 years	149 (23%)
19-22 years	361 (56%)
23-25 years	131 (20%)
Gender	
Female	347 (54%)
Male	294 (46%)
Marital status	
Married	59 (9.2%)
Unmarried	582 (91%)
Educational level	
Illiterate	2 (0.3%)
Middle school	11 (1.7%)
High school	238 (37%)
Diploma	23 (3.6%)
Bachelor's	353 (55%)
Master's/PhD	14 (2.2%)
Residence	
Al-Aqeeq	37 (5.8%)
Al-Baha city	269 (42%)
Al-Hajra	5 (0.8%)
Al-Mandaq	20 (3.1%)
Al-Mikwah	49 (7.6%)
Al-Qara	18 (2.8%)
Baljurashi	76 (12%)
Banni Hassan	31 (4.8%)
Ghamed Al-zinad	98 (15%)
Others	22 (3.4%)
Qelwah	16 (2.5%)

Approximately 70.2% (n=641) of the participants either currently had or had experienced acne. Among them, 65% (n=292) had acne between the ages of 13-16, with the majority experiencing it on their faces (92%, n=415) and backs (40%, n=180). The majority of the participants assessed their acne as moderate (65%, n=292), noted the presence of scars after having acne (60%, n=269), and reported a family history of acne (59%, n=266). In terms of treatment behavior, 36% (n=160), 59% (n=266), and 41% (n=186) mentioned visiting a physician, a pharmacy, or using herbs, respectively, to address their acne, as indicated in Table 2.

**Table 2:** Characteristics of participants with acne (N=450)

Characteristic	n (%)
What is the age of onset of your acne?	
Less than 13	32 (7.1%)
13-16	292 (65%)
17 and above	126 (28%)
Areas affected by acne	
Face	415 (92%)
Chest	86 (19%)
Back	180 (40%)
Others	15 (3.3%)
How would you rate your acne?	
Mild	116 (26%)
Moderate	292 (65%)
Severe	42 (9.3%)
Did your acne leave any scars?	
No	181 (40%)
Yes	269 (60%)
Did any member of your family suffer from acne?	
No	59 (13%)
Yes	266 (59%)
I don’t know	125 (28%)
Have you visited a physician regarding your acne in the past?	
No	290 (64%)
Yes	160 (36%)
Have you visited a pharmacy and got medicine without a prescription to treat your acne?	
No	184 (41%)
Yes	266 (59%)
Have you used any herbs to treat your acne?	
No	264 (59%)
Yes	186 (41%)

The likelihood of experiencing acne was higher among individuals aged 23-25 years, females, and those with a bachelor's degree (p-value < 0.05), as detailed in Table 3.

**Table 3:** Association between experiencing acne and demographic characteristics of the participants ^1^n (%); ^2^Pearson's chi-squared test, Fisher's exact test

Characteristic	Experiencing acne			p-value^2^
	No, N = 191^1^		Yes, N = 450^1^	
Age				0.003
15-18 years	61 (32%)	88 (20%)		
19-22 years	96 (50%)	265 (59%)		
23-25 years	34 (18%)	97 (22%)		
Gender				0.008
Female	88 (46%)	259 (58%)		
Male	103 (54%)	191 (42%)		
Marital status				0.10
Married	12 (6.3%)	47 (10%)		
Unmarried	179 (94%)	403 (90%)		
Educational level				<0.001
Illiterate	1 (0.5%)	1 (0.2%)		
Middle school	5 (2.6%)	6 (1.3%)		
High school	94 (49%)	144 (32%)		
Diploma	9 (4.7%)	14 (3.1%)		
Bachelor's	78 (41%)	275 (61%)		
Master's/PhD	4 (2.1%)	10 (2.2%)		

Severe acne was associated with having a higher risk of low mood and low self-esteem (p-value < 0.001). In comparison, moderately severe acne was linked to a higher risk of avoiding attending social events (p-value = 0.007) and affecting important aspects of life (p-value < 0.001), as highlighted in Table 4.

**Table 4:** Association between acne severity and quality of life ^1^n (%); ^2^Pearson's chi-squared test

	Severity of acne			
Characteristic	Mild, N = 1161	Moderate, N = 2921	Severe, N = 421	p-value2
Low mood				<0.001
No	90 (78%)	165 (57%)	17 (40%)	
Yes	26 (22%)	127 (43%)	25 (60%)	
Low self-esteem				<0.001
No	91 (78%)	184 (63%)	20 (48%)	
Yes	25 (22%)	108 (37%)	22 (52%)	
Avoid attending social events				0.007
No	100 (86%)	214 (73%)	28 (67%)	
Yes	16 (14%)	78 (27%)	14 (33%)	
Effect on important areas of life				<0.001
No	103 (89%)	213 (73%)	27 (64%)	
Yes	13 (11%)	79 (27%)	15 (36%)	
None of the above				<0.001
No	45 (39%)	189 (65%)	33 (79%)	
Yes	71 (61%)	103 (35%)	9 (21%)	

In terms of factors influencing treatment behavior, consulting a physician was associated with individuals aged 19-22 years, females, unmarried individuals, and those with a bachelor's degree. Similarly, seeking assistance from a pharmacy was linked to the same demographics: those aged 19-22 years, females, unmarried individuals, and those with a bachelor's degree. Additionally, the use of herbs showed an association with being female (p-value = 0.001), as presented in Table 5.

**Table 5:** Association between treatment behavior towards acne and demographic characteristics ^1^n (%); ^2^Pearson's chi-squared test, Fisher's exact test

	Visiting a physician			Visiting a pharmacy			Using herbals		
Characteristic	No, N = 290^1^	Yes, N = 160^1^	p-value^2^	No, N = 184^1^	Yes, N = 266^1^	p-value^2^	No, N = 264^1^	Yes, N = 186^1^	p-value^2^
Age			0.012			0.025			>0.9
15-18 years	64 (22%)	24 (15%)		43 (23%)	45 (17%)		52 (20%)	36 (19%)	
19-22 years	175 (60%)	90 (56%)		112 (61%)	153 (58%)		156 (59%)	109 (59%)	
23-25 years	51 (18%)	46 (29%)		29 (16%)	68 (26%)		56 (21%)	41 (22%)	
Gender			0.010			<0.001			<0.001
Female	154 (53%)	105 (66%)		80 (43%)	179 (67%)		125 (47%)	134 (72%)	
Male	136 (47%)	55 (34%)		104 (57%)	87 (33%)		139 (53%)	52 (28%)	
Marital status			0.008			0.024			0.081
Married	22 (7.6%)	25 (16%)		12 (6.5%)	35 (13%)		22 (8.3%)	25 (13%)	
Unmarried	268 (92%)	135 (84%)		172 (93%)	231 (87%)		242 (92%)	161 (87%)	
Educational level			0.007			0.011			0.051
Illiterate	1 (0.3%)	0 (0%)		1 (0.5%)	0 (0%)		1 (0.4%)	0 (0%)	
Middle school	5 (1.7%)	1 (0.6%)		5 (2.7%)	1 (0.4%)		4 (1.5%)	2 (1.1%)	
High school	108 (37%)	36 (23%)		71 (39%)	73 (27%)		86 (33%)	58 (31%)	
Diploma	8 (2.8%)	6 (3.8%)		4 (2.2%)	10 (3.8%)		5 (1.9%)	9 (4.8%)	
Bachelor's	164 (57%)	111 (69%)		99 (54%)	176 (66%)		166 (63%)	109 (59%)	
Master's/PhD	4 (1.4%)	6 (3.8%)		4 (2.2%)	6 (2.3%)		2 (0.8%)	8 (4.3%)	

**Figure 1:**
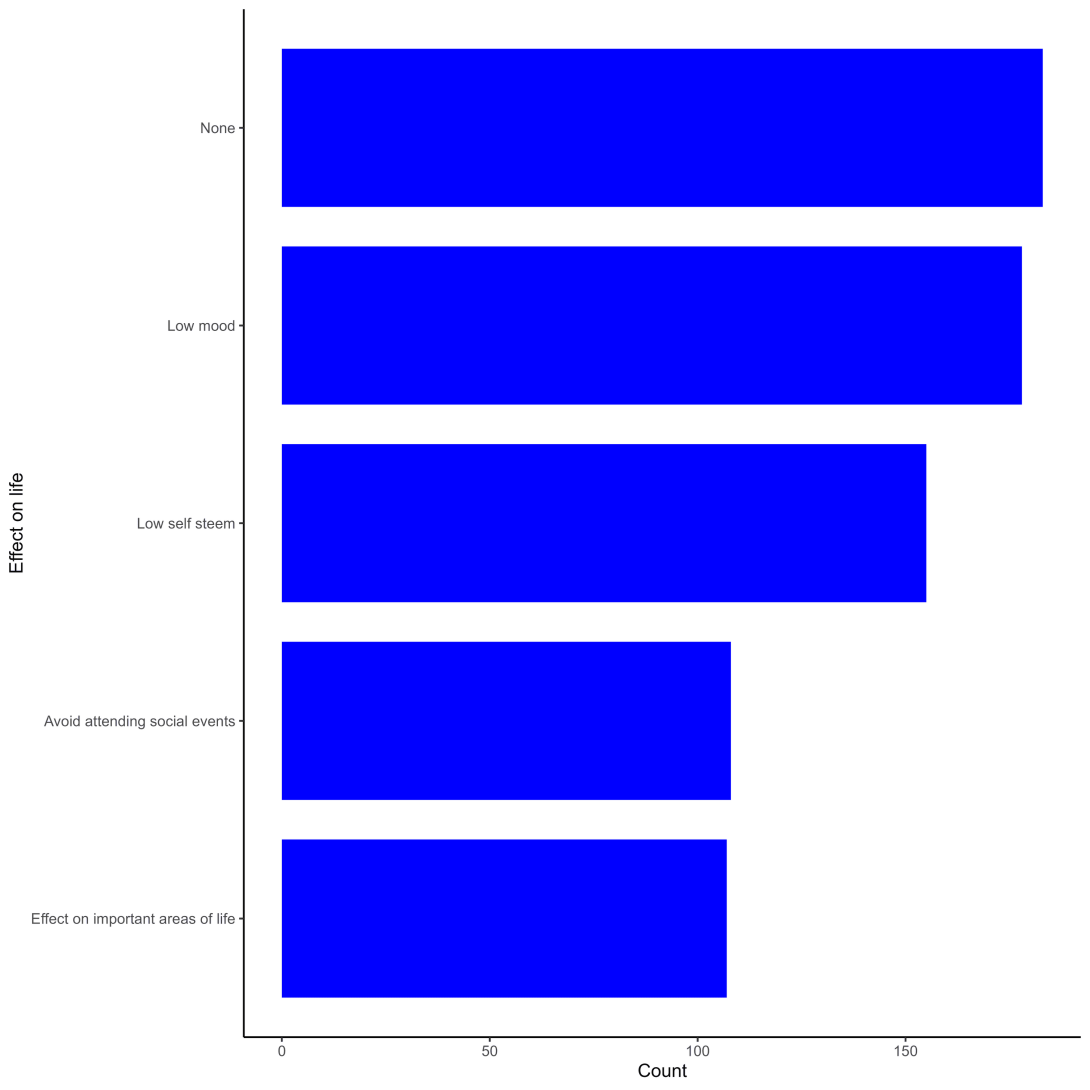
Participants' perception of the impact of acne on quality of life

**Figure 2:**
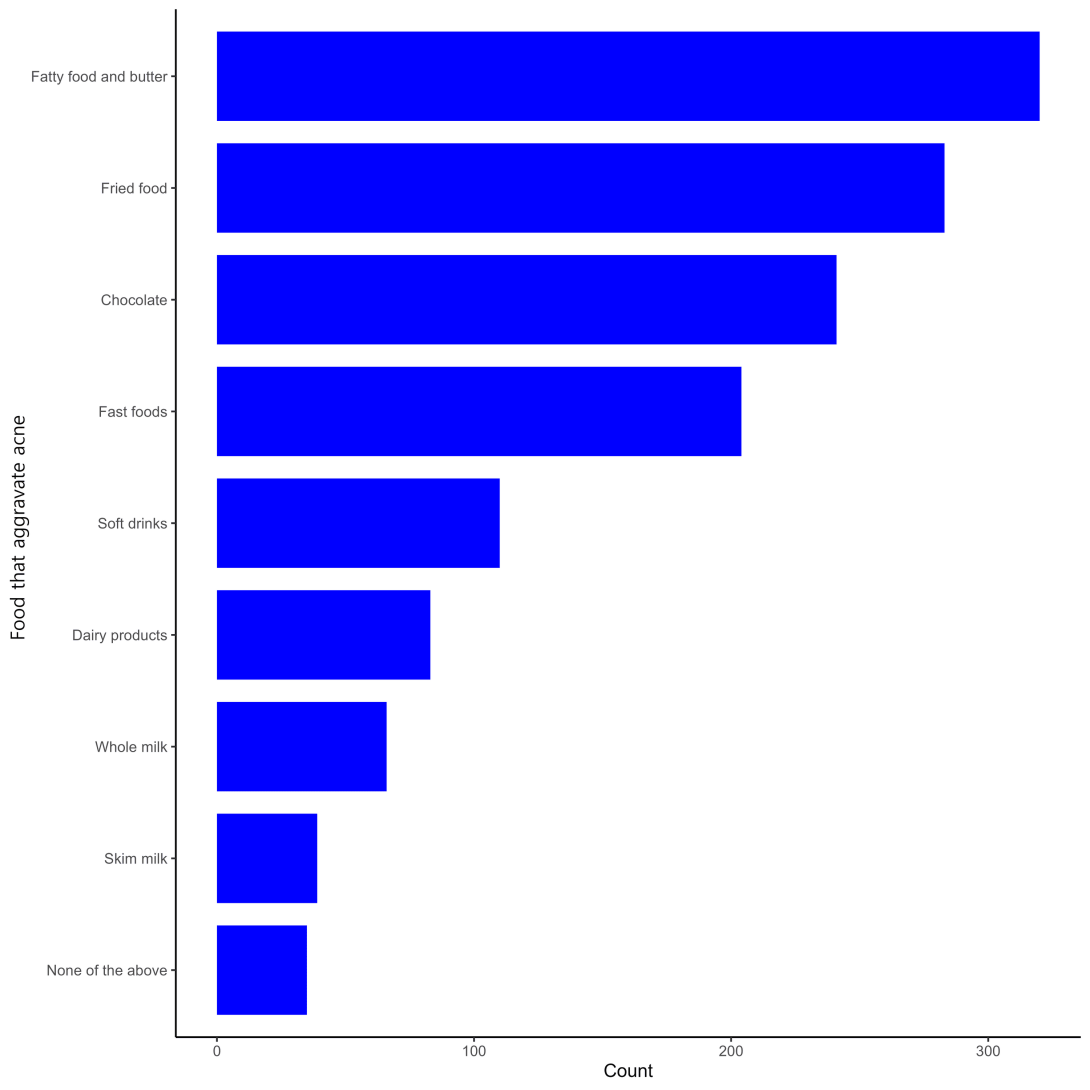
Participants' perception of foods that exacerbate acne

**Figure 3:**
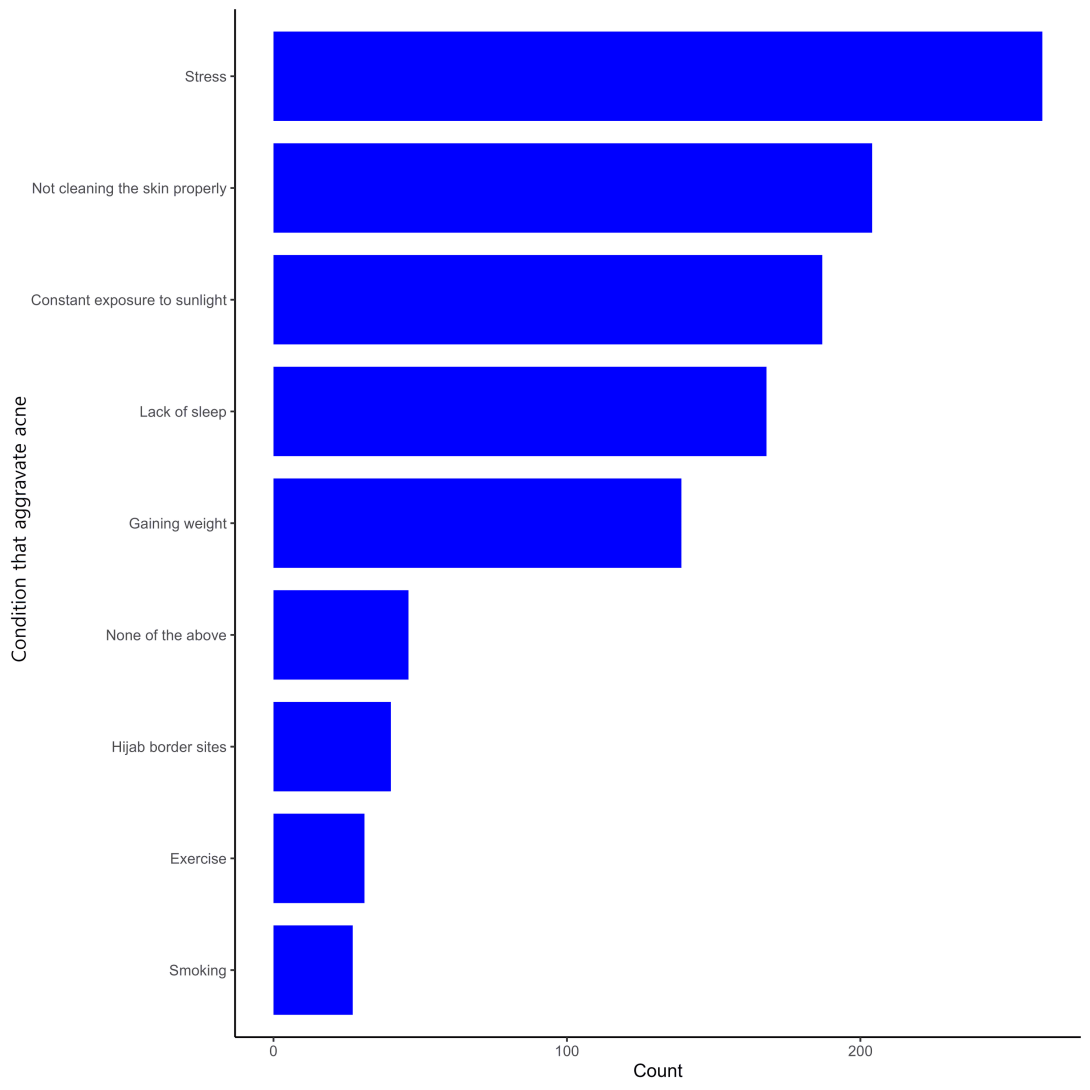
Participants' perception of conditions that exacerbate acne

Most participants reported having low mood (28%) and low self-esteem (24%), as shown in Figure 1. The majority of the participants identified fatty food and butter (50%), fried food (44%), chocolate (38%), and fast food (32%) as major contributors to the exacerbation of acne, as demonstrated in Figure 2. The majority of the participants experiencing acne identified stress (41%), inadequate skin cleaning (32%), prolonged sun exposure (29%), insufficient sleep (26%), and weight gain (22%) as factors contributing to the worsening of their acne, as highlighted in Figure 3.

## Discussion

We conducted this study in order to obtain the prevalence of acne vulgaris in Al-Baha. In 2013, a study in Saudi Arabia stated that acne is prevalent among the Saudi population at a rate of 64.5% [15]. In two earlier studies focusing on students, the prevalence of acne was 56.2% and 55.5%, respectively [5,16]. In our research, a significant proportion (70.2%, n=641) reported either currently having or having experienced acne. Our study specifically focused on individuals aged 15 to 25. This narrower age range might account for the difference in the reported prevalence rates.

We anticipated that there would be a notable difference in the prevalence of acne vulgaris across different age groups. We expected that the younger age groups would exhibit a higher proportion of acne compared to older age groups. This is because acne vulgaris is known to be more prevalent among adolescents, primarily due to the hormonal changes that take place during the teenage years [17]. In our study, we found that individuals aged 19-22 accounted for the highest percentage, with 59% (n=450) reporting acne. This was followed by participants aged 23-25, comprising 22% (n=450) with acne, and those aged 15-18, accounting for 20% (n=450).

Regarding the location of acne, the majority of participants reported it on their faces (92%, n=450), followed by the back (40%, n=450), the chest (19%, n=450), and other areas (3.3%, n=450). Similar results were found in Riyadh, the face was affected by acne in all patients of their study [18]. In addition, another study found that 56.3% of participants had acne in more than one area on the face and 41.7% of students had truncal acne [14].

When participants were asked to assess the severity of their acne, the majority categorized it as moderate (65%, n=450), while 26% (n=450) considered it mild, and 9.3% (n=450) described it as severe. This goes in line with a study conducted in Makkah, Saudi Arabia, showing that the majority of female teenagers had acne with moderate severity (38.9%) followed by mild acne (30%), severe acne (26.6%), and very severe acne (4.5%) [19]. In contrast to our study, a separate study conducted in Jeddah revealed contradictory findings regarding the severity of acne among female medical students. According to their findings, the most common severity levels reported were 68.1% with mild acne, 26.0% with moderate acne, and 5.9% with severe acne [20]. The variations observed between the studies could be attributed to geographical differences, as well as the fact that these studies specifically focused on female participants and did not include males. Also, 60% (n=450) of participants in our study reported the presence of scars after experiencing acne, while the prevalence of scars in another study was 71.1% [18].

Severe acne was found to be associated with a higher risk of low mood and low self-esteem, with 60% (n=42) reporting such symptoms. Similar research conducted in Jordan revealed a strong correlation between the severity of acne and low self-esteem as well as how it affected body image; however, the degree of acne had no effect on daily activities or going to social gatherings [21].

In terms of treatment behaviors, 59% of participants (n=450) reported using treatment without a prescription, 41% (n=450) used herbs, and 36% (n=450) sought dermatological advice. A similar study found that approximately 35.8% of students utilized non-prescription acne treatments, indicating that these treatments are likely over-the-counter medications [14].

Regarding factors influencing treatment behavior, consulting a physician was more prevalent among individuals aged 19-22 years (56%, n=160). Individuals in the age range of 19-22 years are more likely to seek medical advice due to higher awareness and concern about their health compared to other age groups. Females (66%, n=160) were more likely to consult a physician compared to males (34%, n=160), due to societal and cultural factors that prioritize their health and well-being; also, there is a similar study showing it may represent an increased awareness in women seeking treatment as compared to men [22]. Furthermore, participants with a bachelor’s degree demonstrated a higher likelihood (69%, n=160) of consulting physicians, because they may have a better understanding of the importance of seeking professional medical advice and are more likely to do so.

Additionally, participants identified specific dietary factors that they believed contributed to the exacerbation of acne. The majority (50%) cited fatty food and butter, followed by fried food (44%), chocolate (38%), and fast food (32%). According to a study, a significant portion of the Saudi population holds the belief that hormonal disturbances are the primary cause of acne vulgaris. This perception is followed by the belief that diet plays a substantial role in contributing to acne vulgaris. The majority of the population surveyed in the study expressed these views regarding the causes of acne [20].

The majority of participants experiencing acne identified stress (41%), inadequate skin cleaning (32%), prolonged sun exposure (29%), insufficient sleep (26%), and weight gain (22%) as factors contributing to the worsening of their acne. A study indicates that stress is identified as the most influential factor in predicting the occurrence of acne. The study suggests that when individuals experience stress, it can trigger various physiological mechanisms within the body, such as activating the immune, endocrine, and nervous systems. These mechanisms may contribute to the development or exacerbation of acne [20].

Limitations

Several limitations have been noted. Firstly, the questionnaire lacks reliability and validity assessments. Secondly, there was no inquiry made to differentiate between self-reported diagnoses and those verified by healthcare professionals. Furthermore, encountering low response rates in specific areas such as Al-Hajra, Al-Qara, and Al-Mandeq restricts the generalizability of the study’s findings to these regions. To address this issue, targeted outreach strategies have been implemented, including increased community engagement and incentives for data collectors to encourage greater participation from these underrepresented areas.

Recommendations

Based on the participants’ feedback, there is a need for community-based health awareness programs focused on acne to enhance awareness and improve perceptions and beliefs among acne patients. Further research is necessary to identify aggravating factors, assess treatment compliance, and evaluate the impact of acne vulgaris on the quality of life among the Al-Baha population.

## Conclusions

Among the participants, a significant proportion (70.2%, n=641) reported either currently having or having experienced acne. Acne vulgaris was found to be largely prevalent in adolescents and young adults. Therefore, we should increase efforts to raise awareness of acne vulgaris, its treatment, and its complications. Moreover, by clinically evaluating the participants, further research should ascertain the prevalence in a more objective manner.

## Appendices

Instructions

Dear reader,

We are a research team from the faculty of medicine at Al-Baha University inviting you to participate in a research study about the epidemiology of acne vulgaris in young adults and adolescents in Al-Baha, Saudi Arabia.

Participation in this study is completely voluntary and any information you share with us will be kept completely confidential and used only for research purposes.

Please note that you must be between the ages of 15 and 25 to participate in this study.

I agree to participate in this research study. I understand the nature of this study and I am participating voluntarily.

¨ Yes

¨ No

Sociodemographic data

1. Your Age

¨  15-18

¨  19-22

¨  23-25

2. Your Gender

¨  Male

¨  Female

3. Current marital status

¨  Married

¨  Single

4. Level of education

¨  Illiterate

¨  Middle school

¨  High school

¨  Diploma

¨  Bachelor's

¨  Master's

¨  PhD

5. In which governate of Al-Baha do you live?

¨  Al-Baha city

¨  Baljurashi

¨  Al-Mandaq

¨  Al-Mikwah

¨  Al-Aqeeq

¨  Qelwah

¨  Al-Qara

¨  Banni Hassan

¨  Ghamed Al-zinad

¨  Al-Hajra

Acne and its characteristics

6. Do you have acne currently or did you have acne in the past?

¨  Yes

¨  No (you can skip the rest of the questionnaire by pressing "send")

7. What is the age of onset of your acne?

¨  <13

¨  13-14

¨  15-16

¨  17-18

¨  >18

8. Choose one or more of the following sites on which acne present:

¨  Face

¨  Chest

¨  Back

¨  Others

9. How would you rate your acne?

¨  Mild

¨  Moderate

¨  Severe

10. Did your acne leave any scars?

¨  Yes

¨  No

Associated factors

11. Did any member of your family suffer from acne?

(You can select more than one answer)

¨  No one

¨  Father

¨  Mother

¨  Siblings

12. Which of the following conditions do you think aggravated your acne?

(You can select more than one answer)

¨  Not cleaning the skin properly

¨  Constant exposure to sunlight

¨  Exercise

¨  Lack of sleep

¨  Gaining weight

¨  Stress

¨  Smoking

¨  Hijab border sites

¨  Menstrual cycle

¨  None of the above

13. Which of the following foods do you think aggravated your acne?

(You can select more than one answer)

¨  Fatty food and butter

¨  Fried food

¨  Chocolate

¨  Dairy products

¨  Skim milk

¨  Whole milk

¨  Soft drinks

¨  Fast foods

¨  None of the above

Treatment behaviors

14. Have you visited a physician regarding your acne in the past?

¨  Yes

¨  No

15. Have you visited a pharmacy and got medicine without a prescription to treat your acne?

¨  Yes

¨  No

16. Have you used any herbals to treat your acne?

¨  Yes

¨  No

Impact of acne on quality of life and the psychology of the patient

17. Choose one or more of the following effects acne had on you:

¨  Low self-esteem

¨  Low mood (i.e., depression)

¨  Avoid attending social events

¨  Effect on important areas of life (e.g., work, school)

¨  None of the above
